# Skin Microvascular Dysfunction in Type 2 Diabetes Mellitus Using Laser Speckle Contrast Analysis and Association with Carotid Intima-Media Thickness

**DOI:** 10.3390/jcm13164957

**Published:** 2024-08-22

**Authors:** Stamatina Lamprou, Nikolaos Koletsos, Ioanna Zografou, Antonios Lazaridis, Gesthimani Mintziori, Christina Maria Trakatelli, Vasilios Kotsis, Eugenia Gkaliagkousi, Michael Doumas, Areti Triantafyllou

**Affiliations:** 1 Third Department of Internal Medicine, Papageorgiou General Hospital, Aristotle University of Thessaloniki, 56429 Thessaloniki, Greece; stamatin@auth.gr (S.L.); alazarif@auth.gr (A.L.); ctrak@auth.gr (C.M.T.); vkotsis@auth.gr (V.K.); egkaliagkousi@auth.gr (E.G.); artriant@auth.gr (A.T.); 2 Second Propedeutic Department of Internal Medicine, Hippokration General Hospital, Aristotle University of Thessaloniki, 54642 Thessaloniki, Greece; ioannazo@yahoo.gr (I.Z.); doumasm@auth.gr (M.D.); 3 Unit of Reproductive Endocrinology, 1st Department of Obstetrics and Gynecology, Papageorgiou General Hospital, Aristotle University of Thessaloniki, 56429 Thessaloniki, Greece; gefsi@auth.gr

**Keywords:** diabetes mellitus, cardiovascular disease, intima-media thickness, laser speckle, microcirculation, microvascular dysfunction

## Abstract

**Background:** It is established that diabetes mellitus (DM) is characterized by increased cardiovascular risk associated with subclinical atherosclerosis as well as microvascular alterations. Laser speckle contrast analysis (LASCA) is an innovative, non-invasive method for assessing skin microvascular function. **Objectives:** We sought to assess skin microvascular function in patients with type 2 DM and matched controls. **Methods:** Consecutive patients with DM and individuals matched for age, sex and BMI were included in the study. Skin microvascular perfusion was assessed, using LASCA, during baseline, a 5 min occlusion period and a 5 min reperfusion period. Carotid intima-media thickness (cIMT) was measured as a surrogate marker of macrocirculation. **Results:** In total, 18 patients with DM and 22 in the control group were enrolled. No statistically significant differences were observed in baseline flux, peak flux and percentage decrease during arterial occlusion. During reperfusion, individuals with DM exhibited a smaller peak magnitude compared to controls (147.0 ± 64.7% vs. 189.4 ± 46.0%, respectively; *p* < 0.05). Moreover, cIMT was higher in patients with DM compared to controls (0.68 ± 0.09 mm vs. 0.60 ± 0.08 mm, respectively, *p* < 0.01) and was negatively correlated with skin microvascular reactivity in the univariate analysis. In the multivariate analysis, glucose and office systolic blood pressure levels remained significant predictors of microvascular reactivity. **Conclusions:** Our study shows that patients with type 2 DM exhibit impaired skin microvascular function compared to controls. Furthermore, glucose levels and blood pressure play a key role in microvascular dysfunction. However, additional studies are needed to address the clinical significance of early microvascular changes in DM.

## 1. Introduction

It is well known that diabetes mellitus (DM) is a significant risk factor for cardiovascular disease (CVD) [1]. Data show that people with DM have a 2–4-fold higher risk of mortality due to heart disease and stroke than those without diabetes [2]. It has also been demonstrated that people with DM have an equal risk for coronary heart disease as non-diabetic people with a prior myocardial infarction [3]. Classic risk factors such as obesity, lack of glycemic control, hypertension and dyslipidemia are the main contributors to the occurrence of CVD in subjects with DM. Moreover, oxidative stress, inflammation and endothelial dysfunction are proven determinant factors for macro- and microvascular damage [2,4].

Macro- as well as microvascular complications are the primary cause of mortality and morbidity in individuals with DM. Macrovascular complications include coronary artery disease, peripheral arterial disease (PAD) and stroke [5]. DM is accompanied by an increased risk of the above-mentioned conditions independently of traditional CV risk factors such as hypertension or dyslipidemia [6]. It is proven that hyperglycemia is associated with increased arterial stiffness. Therefore, the induced structural changes in the vascular wall lead to atherosclerosis, which is the main contributor to the pathogenesis of CVD [7].

Carotid artery ultrasonography is a non-invasive and inexpensive method, which allows carotid imaging in real time. The carotid intima-media thickness (cIMT) is measured between the intimal–luminal and the medial–adventitial interfaces of the vessel wall, represented as a double-line density on an ultrasound image. Large epidemiological studies have shown that cIMT can be used as a predictor of future CV disease [8]. Besides CV events, a recent meta-analysis emphasizes the positive association of increased cIMT with other macro- and microvascular complications of DM [9]. Interestingly, Brohall et al. showed that subjects with DM had approximately a 0.13 mm thicker carotid artery IMT as compared to the control group. In clinical terms, this difference in cIMT can be interpreted as if individuals with DM were more than 10 years older than the control groups [10]. Taking those data into account, carotid ultrasound has been established as a valuable tool for the early detection of long-term complications of diabetes.

As mentioned above, microvascular complications are common in patients with DM and are the leading cause of morbidity. Diabetic microangiopathy includes retinopathy, nephropathy and neuropathy [11]. Briefly, the term microcirculation refers to the circulation in vessels <150 µm in diameter, comprising the small arteries and veins, as well as the capillaries [12]. Diabetic retinopathy (DR) is the most common microvascular complication of DM and remains the main cause of blindness [13]. Data note that the severity of DR can be used as a predictive factor for the occurrence of CVD [14,15,16], which may be due to the similar pathophysiological pathways that DR and CVD share [16].

Of note, microvascular alterations can be detected even in the early stages of CVD, long before the observation of apparent clinical manifestations, and in several vascular beds. Skin microcirculation is easily accessible and plays a crucial role in the assessment of microvascular function. The relationship between microvascular dysfunction and CV risk has been demonstrated by studying skin microvascular reactivity [17,18]. Indeed, various non-invasive techniques have recently emerged to evaluate skin microvascular reactivity.

Laser speckle contrast analysis (LASCA) is a non-invasive laser technique for assessing skin microvascular function. LASCA represents an innovative evolution of the older laser Doppler flowmetry methods, thus providing better spatial resolution. It allows assessment of microvascular perfusion in a larger tissue area in real time and with higher reproducibility. Briefly, the main principles of its function are based on the speckle phenomenon to create dynamic two-dimensional maps of skin microvascular perfusion and visualize blood flow with high spatial and temporal resolution [19,20]. The mapping of microcirculation concerns the upper layer of skin at depths of ~300 μm, provided that a laser wavelength of 780 nm is used [21]. Microvascular responsiveness can be evaluated via numerous stimuli, such as iontophoresis with acetylcholine, thermal challenges or post-ischemic forearm skin reactive hyperemia [21,22].

An ever-increasing number of studies regarding the application of LASCA in clinical practice have been published in recent years. The majority of data included patients with connective tissue diseases [23,24,25,26,27]. Nevertheless, lately, it has been applied in patients at increased CV risk [20,28,29]. On the other hand, few studies have emerged in the field of DM. Matheus et al. assessed the microvascular reactivity in patients with type 1 DM using LASCA coupled with various stimuli (iontophoresis with acetylcholine; post-occlusive reactive hyperemia—PORH). This study showed that microvascular reactivity was significantly affected compared to healthy controls [30]. In the same way, Fuchs et al. demonstrated the presence of dermal microvascular dysfunction as assessed by LASCA or laser Doppler in a population mainly comprising individuals with type 1 DM compared to a healthy group, using local thermal hyperemia as a stimuli [31]. Canto et al. showed that women, more than men, with type 2 DM, who present skin microvascular dysfunction, are at increased risk of developing heart failure with preserved ejection fraction [32]. Moreover, the LASCA technique has been used for the assessment of microvascular dysfunction in PAD [33] and in foot ulcers [34,35] in subjects with long-standing DM. However, to our knowledge, no previous study has examined skin microvascular function using LASCA in patients with type 2 DM without established CV disease nor significant complications as compared to controls.

Given the above, the aims of the present study were (i) to evaluate the presence of microvascular alterations using LASCA in patients with type 2 DM, (ii) to identify possible factors associated with microvascular response, and (iii) to examine possible associations between micro- and macrocirculation using IMT, an established marker of macrocirculation.

## 2. Materials and Methods

### 2.1. Participants

This study was performed between October 2020 and June 2023. Patients from the outpatient clinic, with an established diagnosis of DM according to the American Diabetes Association (ADA) criteria, were included in the study. In more detail, the diagnosis of DM was made if one of the following existed: (1) elevated fasting plasma glucose of >126 mg/dL or elevated random plasma glucose of >200 mg/dL, confirmed by repeated testing on another day, (2) 2 h plasma glucose level >200 mg/dL during a 75 g oral glucose tolerance test, or (3) glycated hemoglobin (HbA1c) ≥6.5% [36]. Both patients with a previous DM diagnosis and newly diagnosed individuals were recruited. Patients with type 1 DM, a known history of established cardiovascular disease or other significant comorbidities were excluded from the study. The control group consisted of individuals matched for age, sex and body mass index (BMI), and were recruited from both the outpatient clinic and the community. All individuals were over 18 years old and gave their written consent prior to study enrollment. The study protocol was conducted in accordance with the principles of the Declaration of Helsinki and was approved by the institutional review board committee [37].

### 2.2. Clinical Assessment

After obtaining a detailed medical history, a physical examination was performed. BMI was calculated for each participant by dividing body weight in kilograms by the square of height in meters. Office blood pressure (BP) was measured three times in each participant, in the sitting position, with a 2 min interval between each measurement, according to a standard protocol [38]. The average of the last two measurements was considered as the office BP and all measurements were completed using a validated oscillometric device (Microlife AG Swiss Corporation, Widnau, Switzerland) with the appropriate cuff size. Hypertension was defined as office systolic and/or diastolic BP ≥ 140/90 mmHg, according to the 2018 guidelines from the European Society of Hypertension and/or current antihypertensive medication [38]. Moreover, blood samples were derived from all participants after an overnight fast. Plasma glucose levels and lipid profile (total cholesterol, low-density lipoprotein, high-density lipoprotein and triglycerides) were determined using routine laboratory techniques (Architect c16000, Abbott). HbA1c was quantified using high-performance liquid chromatography (Hb NEXT, Lawrenceville, GA, USA, Menarini Diagnostics, Athens, Greece) in whole blood samples.

### 2.3. Macrovascular Assessment

Macrovascular assessment included the evaluation of cIMT. Longitudinal images of the common carotid arteries were captured in the supine position using an ultrasound device (Aloka Pro Sound A7, Ultrasound System, Tokyo, Japan), and cIMT was measured as the distance between the intimal–luminal and the medial–adventitial interfaces of the vessel represented as a double-line density on an ultrasound image [8]. cIMT was calculated as the mean of three consecutive measurements, and the average cIMT of the left and right carotid artery was used for the analysis.

### 2.4. Microvascular Assessment

Participants were instructed to refrain from smoking or consuming coffee, tea and alcohol for at least 4 h before their visit to our laboratory. All measurements were performed in the supine position and in a separate, quiet, temperature-controlled room (23.5 °C ± 1 °C) with low ambient light and no significant air movement.

Cutaneous blood flow was recorded using a LASCA device (PeriCam PSI NR System, Perimed, Järfälla, Sweden). The device uses a laser wavelength of 785 mm, and the skin penetration depth is ~300 μm [29]. The distance between the laser head and the forearm skin surface in all recordings was fixed at 15 ± 1 cm, as suggested by the manufacturer. A vacuum cushion was applied to each patient’s right arm in order to immobilize it and reduce arm movement and, thus, motion artefacts [22]. The temperature of the studied skin surface was measured at the beginning of each measurement with a non-contact thermometer (Microlife AG Swiss Corporation, Widnau, Switzerland). BP was also assessed on the left arm while participants were lying supine using the same device as mentioned earlier.

After a 20 min acclimatization period, recording of skin microvascular perfusion was started. In our study, we used the PORH protocol, one of the most widely used methods to assess reactivity. It represents a temporary increase in blood flow following the release of a previously occluded artery [22,39,40]. More specifically, the PORH protocol included a 3 min baseline recording and a 5 min period of brachial artery occlusion by inflating a pressure cuff at suprasystolic levels (250 mmHg) to occlude skin blood perfusion. Then, after rapid deflation of the cuff, a 5 min recovery period followed [23,29]. Two circular skin sites (regions of interest, ROIs) were randomly chosen on the surface of the examined forearm. Each ROI had a 10 mm radius and the average blood perfusion of the two ROIs per testing period was used in the analysis. Areas with visible veins, tattoos, scars, skin pigmentation and hair growth were avoided.

Data analysis was performed using the manufacturer’s software (PIMSoft, Perimed, Järfälla, Sweden) and the recorded blood flux was expressed in arbitrary perfusion units (PU). Mean perfusion during the baseline period (baseline flux, PU), percentage decrease in perfusion from baseline to maximum occlusion (%), mean perfusion at the maximum response of the post-occlusive period (peak flux, PU), and percentage increase in perfusion from baseline to the maximum post-occlusive response (peak magnitude, %) were reported.

### 2.5. Statistical Analysis

The sample size calculation was based on available data from the literature on individuals with type 1 diabetes mellitus [30] and studies conducted in our department [23,24,29]. Sample size calculation was performed a priori, using G*Power software (Version 3.1, Heinrich-Heine University, Düsseldorf, Germany). Assuming type I error, α = 0.05 and 1 − β = 0.8 statistical power, the estimated total sample size required to detect differences in microvascular response was 38 individuals.

Statistical analysis was performed using SPSS software (IBM SPSS Statistics 25.0, Chicago, IL, USA). Normally distributed variables are described as mean ± standard deviation, while non-normally distributed are described as median ± interquartile range. Differences among groups were examined by the independent samples t-tests or the non-parametric Mann–Whitney test based on the normality of the distribution. Qualitative variables were compared by the χ^2^ test or Fisher’s exact test when necessary and results were expressed as percentages. To identify possible associations within the whole population, Pearson’s or Spearman’s correlations were used depending on the normality of distribution. Subsequently, multiple linear regression analysis was performed to identify possible predicting factors of microvascular reactivity, after adjustment for other parameters. In the multivariate analysis, variables that either differed between the two groups or significantly correlated with microvascular reactivity in the univariate analysis were included. In cases of originally non-normally distributed variables, logarithmic transformation was performed. A *p*-value < 0.05 was considered statistically significant.

## 3. Results

In total, 40 individuals (mean age 55.7 ± 8.0 years) were included in the study: 18 patients with DM and 22 in the control group. No statistically significant differences were observed between the two groups regarding age, sex, BMI or smoking status. The proportion of obese individuals was 47.1% in the DM group and 31.8% in the control group; however, the difference did not reach statistical significance (*p* = 0.332). More participants in the DM group had a history of hypertension (77.8% vs. 31.8%, respectively; *p* < 0.01); however, no differences regarding both office and supine BP before microcirculation assessment were observed between the two groups. As expected, participants in the DM group exhibited significantly higher glucose levels (130.2 ± 48.4 mg/dL vs. 90.5 ± 6.2 mg/dL, respectively; *p* = 0.005) and mean glycosylated hemoglobin in the DM groups was significantly higher as compared to the control group (6.5 ± 1% vs. 5.4 ± 0.2%, respectively; *p* = 0.003). The baseline characteristics of the study population are presented in Table 1.

Median DM duration was 1 (0.4–8.0) year. Of the individuals with DM, five were newly diagnosed (less than 6 months) and were treated only with lifestyle changes (without medications). Among patients with DM under treatment, either alone or in combination, the majority (92.3%) were on metformin, 23.1% insulin, 15.4% sodium glucose cotransporter-2 (SGLT2) inhibitors, 15.4% dipeptidyl peptidase-4 (DPP-4) inhibitors, 7.7% glucagon-like peptide-1 (GLP-1) agonists and 7.7% glitazones. Regarding concomitant treatment, lipid-lowering (52.9% vs. 13.6%) and antihypertensive (72.2% vs. 22.7%) drug use was significantly higher among DM individuals (*p* < 0.1 for both comparisons).

The skin temperature of the examined surface did not differ between the groups at the beginning of the microcirculation assessment (36.0 ± 0.4 °C vs. 35.9 ± 0.2 °C, respectively; *p* = 0.583). Baseline flux, peak flux and percentage decrease in perfusion during arterial occlusion did not differ between the two groups (Table 2).

**Figure 1:**
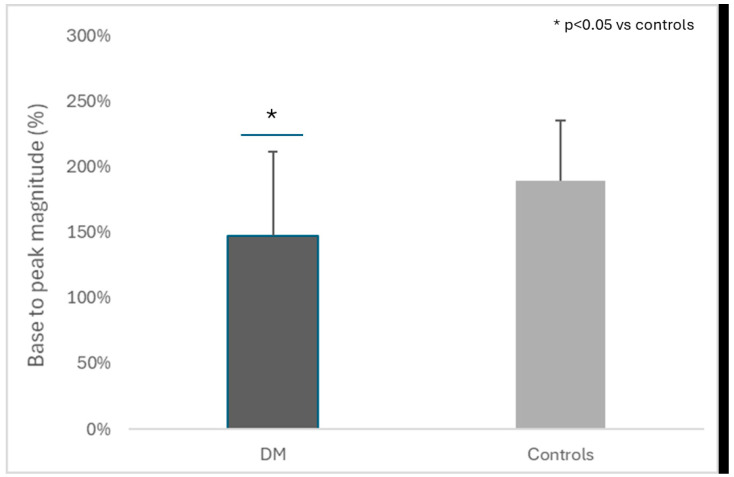
Skin microvascular reactivity as assessed by laser speckle contrast analysis. Comparison of base to peak magnitude following post-occlusive reactive hyperemia in patients with diabetes mellitus and matched controls. DM: diabetes mellitus.

During reperfusion, peak flux increased in both groups. Patients with DM, however, exhibited a smaller peak magnitude (Figure 1) compared to controls (147.0 ± 64.7% vs. 189.4 ± 46.0%, respectively; *p* < 0.05). Moreover, cIMT, even though in the normal limits, was higher in patients with DM compared to controls (0.68 ± 0.09 mm vs. 0.60 ± 0.08 mm, respectively; *p* < 0.01).

**Figure 2:**
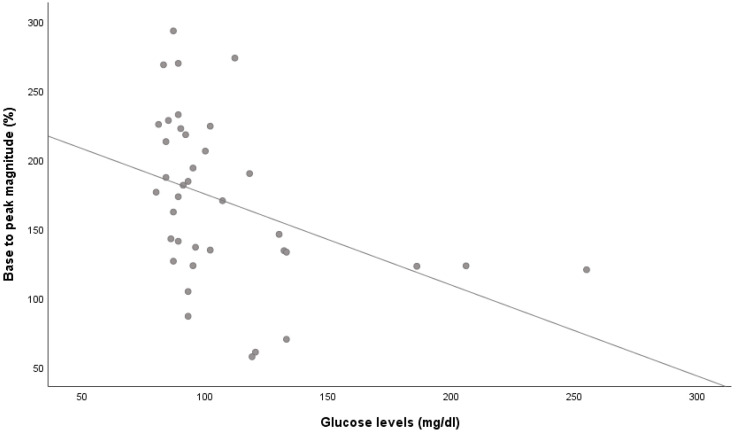
Correlation between skin microcirculation and glucose levels. Skin microvascular reactivity, assessed by laser speckle contrast analysis, correlated with plasma glucose levels.

In the univariate analysis, peak magnitude was inversely correlated with age (r = −0.341, *p* = 0.031), office systolic BP (r = −0.400, *p* = 0.011), glucose levels (r = −0.543, *p* < 0.001, Figure 2) and triglycerides (r = −0.363, *p* = 0.029). In addition, a significant negative association was found between peak magnitude and cIMT (r = −0.388, *p* = 0.016). No significant correlations were observed between the other microvascular-related variables (baseline flux, percentage decrease during occlusion and peak flux) with glucose levels or other baseline characteristics.

In the multivariate analysis, only glucose levels (β = −0.380, *p* = 0.019) and office systolic BP (β = −0.334, *p* = 0.038) remained significant predictors of microvascular reactivity (Table 3), independently of age, triglycerides, IMT and concomitant medication. Glucose levels remained significant predictors of microvascular reactivity, even when the history of DM was included as a binary variable in the analysis (Model 2).

## 4. Discussion

To our knowledge, this is the first study to assess skin microvascular function using the LASCA method in patients with type 2 DM without established CVD or other complications. Our findings showed that patients with DM demonstrate impaired skin microvascular reactivity as compared with individuals matched for sex, age and the presence of other conventional CV risk factors. Interestingly, microvascular reactivity was associated with glucose and BP levels, independently of other parameters. Glucose levels remained significant predictors of microvascular response, even when the history of DM was included as a binary variable in the analysis. It seems that glucose levels (and therefore good glycemic control) may play a more important role in microvascular dysfunction than just DM history. Another finding that merits attention is that microvascular alterations correlated with cΙΜΤ, a well-studied index of macrocirculation (subclinical atherosclerosis), hence supporting the cross-talk between macro- and microcirculation [41]. However, the observed association was no longer significant after adjustment for other parameters. It appears that glucose levels and hemodynamic changes due to BP could be the pathophysiological links for the observed association between microvascular perfusion and subclinical atherosclerosis in DM.

To minimize the systematic error in our study, the two groups did not differ in age, sex or BMI. Similarly, as BP and smoking may influence microvascular reactivity, participants in the two groups had similar BP levels and smoke exposure. Most of the included patients with DM were newly diagnosed, with a median disease duration of 1 year. As expected, lipid-lowering and antihypertensive drug use was significantly higher among DM individuals. This resulted in relatively well-controlled blood pressure levels in patients with DM and lower (although not statistically significant) cholesterol levels as compared to the control group. However, this was not enough to respond to the ischemic period, leading to a significantly lower microvascular reactivity during reperfusion.

The main finding of this study is that patients with DM exhibited lower microvascular reactivity during post-occlusive reperfusion, although the reduction range in blood perfusion during occlusion did not differ between groups. This finding reflects an impairment in skin microvascular function among patients with DM. Moreover, individuals with DM had higher cIMT values, although within the normal range. Microvascular dysfunction has been demonstrated in patients with DM using the LASCA method in previous studies. In accordance with our results, De Matheus et al. showed that skin microvascular response to a stimulus was impaired in patients with type 1 DM as compared to healthy controls. In this study, researchers used both iontophoresis with acetylcholine and PORH as stimuli to assess microvascular reactivity [30]. Similarly, Canto et al. assessed skin microvascular function by LASCA using iontophoresis in individuals with DM and showed that people with impaired skin microvascular function are at increased risk of developing heart failure with preserved ejection fraction. The interesting fact in this study was that women more than men had a higher risk of developing heart failure [32].

It is well known that microvascular dysfunction precedes macrovascular complications, so it has a strong association with CVD. In our study, individuals with DM, compared to controls, had higher cIMT, a recognized index of subclinical atherosclerosis. Our results were in accordance with the meta-analysis of Brohall et al., who estimated that cIMT in DM was about 0.13 mm higher compared to the control group [10].

The development of microvascular disease in DM is the result of several pathophysiological mechanisms including oxidative stress, overproduction of reactive oxygen species (ROS) and the formation of advanced glycation end (AGE) products due to hyperglycemia. An alternative mechanism of vascular damage is the suppression of nitric oxide (NO) [17,42], which plays multiple roles in vasculature, such as vasodilation or the formation of atherosclerotic plaque induced by vascular smooth muscle proliferation [43,44].

In the present study, the majority of individuals with DM had, also, a history of hypertension. Available data support that hypertension is common in people with DM type 2 and its prevalence ranges from 50% to 80% in DM type 2 and 30% in DM type 1 [45,46]. The coexistence of DM and hypertension is associated with a higher risk of CVD [47]. It has been demonstrated that insulin resistance and elevated glucose levels promote arterial stiffness, which plays a crucial role in the pathogenesis of hypertension [48]. The activation of the renin–angiotensin–aldosterone system and the sympathetic nervous system, inflammation, oxidative stress and the release of extracellular vesicles (EVs) are some of the underlying mechanisms in the development of hypertension in people with DM type 2 and insulin resistance [49].

Despite the interesting findings reported, our study has some unavoidable limitations. First, its relatively small sample size limits the evaluation of possible risk factors. An additional limitation is the cross-sectional design of the study. Therefore, future prospective studies with larger sample sizes will be beneficial to enhance the power of the present one. Another limitation concerning laser methods is that they provide a measure of relative changes in skin perfusion expressed in arbitrary perfusion units, rather than providing a quantitative index of blood flow in absolute units. Furthermore, insulin was not measured as part of the study protocol. Although hyperinsulinemia and insulin resistance have been proposed to contribute to vascular dysfunction, available studies have conflicting results and their exact role is still to be defined.

## 5. Conclusions

In conclusion, the LASCA is a relatively novel method for assessing skin microvascular function and allows the dynamic analysis of microvasculature non-invasively. It was observed that patients with DM present impaired skin microvascular reactivity compared to controls. Our study findings may suggest that glucose levels (glycemic control) and blood pressure play a fundamental role in microvascular dysfunction. Appropriately designed LASCA studies should be carried out to shed light on the clinical significance of early microvascular changes in DM.

## Figures and Tables

**Table 1:** Baseline characteristics of the study population.

	DM (*n* = 18)	Control (*n* = 22)	*p* Value
Age (years), mean ± S.D.	58.6 ± 7.3	53.8 ± 8.2	0.097
BMI (kg/m^2^), mean ± S.D.	30.1 ± 5.3	27.7 ± 5.6	0.186
Male sex, (%)	50.0	45.5	0.775
Smoking yes, (%)	44.4	36.4	0.604
Hypertension history, yes (%)	77.8	31.8	0.004
Office SBP (mmHg), mean ± S.D.	130.7 ± 18.9	127.1 ± 18.0	0.544
Office DBP (mmHg), mean ± S.D.	81.3 ± 9.1	83.1 ± 11.6	0.592
Office HR (pulses/min), mean ± S.D.	74.1 ± 8.2	68.9 ± 8.5	0.062
Glucose (mg/dL), mean ± S.D.	130.1 ± 48.4	90.5 ± 6.2	0.005
HbA1c (%), mean ± S.D.	6.5 ± 1.0	5.4 ± 0.2	0.003
Total Cholesterol (mg/dL), mean ± S.D.	187.2 ± 51.8	209.3 ± 41.8	0.154
Triglycerides (mg/dL), median (IQR)	128.0 (73)	93.0 (44)	0.096
HDL Cholesterol (mg/dL), mean ± S.D.	40.3 ± 10.7	48.0 ± 12.2	0.050
LDL Cholesterol (mg/dL), mean ± S.D.	116.8 ± 42.2	138.2 ± 37.8	0.109

DM: diabetes mellitus; S.D.: standard deviation; IQR: interquartile range; BMI: body mass index; SBP: systolic blood pressure; DBP: diastolic blood pressure; HR: heart rate; HbA1c: glycosylated hemoglobin; eGFR: estimated glomerular filtration rate; HDL: high-density lipoprotein; LDL: low-density lipoprotein.

**Table 2:** Micro- and macrovascular assessment of the study population.

	DM (*n* = 18)	Control (*n* = 22)	*p* Value
Supine SBP (mmHg), mean ± S.D.	130.8 ± 19.8	124.3 ± 20.4	0.405
Supine DBP (mmHg), mean ± S.D.	80.8 ± 8.9	79.6 ± 13.2	0.773
Baseline flux (PU), mean ± S.D.	41.8 ± 12.6	38.5 ± 8.3	0.328
Baseline to occlusion change (%), median (IQR)	−81.0 ± 6.3	−80.1 ± 9.7	0.728
Peak flux (PU), mean ± S.D.	99.7 ± 29.4	110.4 ± 25.3	0.224
Peak magnitude (%), mean ± S.D.	147.0 ± 64.7	189.4 ± 46.0	0.021
IMT (mm), mean ± S.D.	0.68 ± 0.09	0.60 ± 0.08	0.006

Variables are presented as means ± SD (standard deviation). DM: diabetes mellitus; PU: perfusion units; S.D.: standard deviation; IMT: intima-media thickness.

**Table 3:** Multivariate linear regression analysis for microvascular reactivity in the total population.

Variables	Beta	*p* Value
Model 1, Adjusted R^2^ = 0.263		
Age (years)	0.076	0.742
Office SBP (mmHg)	−0.334	0.038
Glucose levels (mg/dL)	−0.380	0.019
Triglycerides (mg/dL)	−0.076	0.369
Lipid-lowering drug use	−0.190	0.278
Antihypertensive drug use	0.218	0.340
IMT (mm)	−0.146	0.573
Model 2, Adjusted R^2^ = 0.263		
Age (years)	0.076	0.745
Office SBP (mmHg)	−0.334	0.038
Glucose levels (mg/dL)	−0.380	0.019
Triglycerides (mg/dL)	−0.083	0.698
Lipid-lowering drug use	−0.213	0.277
Antihypertensive drug use	0.210	0.371
IMT (mm)	−0.125	0.648
DM history	0.069	0.775

Dependent variable: peak magnitude (%). SBP: systolic blood pressure; DM: diabetes mellitus; IMT: intima-media thickness.

## Data Availability

Data are available upon request from the corresponding author.
